# Temporary establishment of bacteria from indoor plant leaves and soil on human skin

**DOI:** 10.1186/s40793-022-00457-7

**Published:** 2022-12-26

**Authors:** Gwynne Á. Mhuireach, Ashkaan K. Fahimipour, Roo Vandegrift, Mario E. Muscarella, Roxana Hickey, Ashley C. Bateman, Kevin G. Van Den Wymelenberg, Brendan J. M. Bohannan

**Affiliations:** 1 Biology and the Built Environment Center, University of Oregon, Eugene, OR USA; 2 Institute of Ecology and Evolution, University of Oregon, Eugene, OR USA; 3 Department of Biological Sciences, Florida Atlantic University, Boca Raton, FL USA; 4 United States Department of Agriculture, APHIS, PPQ, Miami, FL USA; 5 Institute of Arctic Biology, University of Alaska Fairbanks, Fairbanks, AK USA

**Keywords:** Microbiome, Human skin, 16S, Phyllosphere, Indoor plants, Soil, Potting mix, Microbial transmission

## Abstract

**Background:**

Plants are found in a large percentage of indoor environments, yet the potential for bacteria associated with indoor plant leaves and soil to colonize human skin remains unclear. We report results of experiments in a controlled climate chamber to characterize bacterial communities inhabiting the substrates and leaves of five indoor plant species, and quantify microbial transfer dynamics and residence times on human skin following simulated touch contact events. Controlled bacterial propagule transfer events with soil and leaf donors were applied to the arms of human occupants and repeatedly measured over a 24-h period using 16S rRNA gene amplicon sequencing.

**Results:**

Substrate samples had greater biomass and alpha diversity compared to leaves and baseline skin bacterial communities, as well as dissimilar taxonomic compositions. Despite these differences in donor community diversity and biomass, we observed repeatable patterns in the dynamics of transfer events. Recipient human skin bacterial communities increased in alpha diversity and became more similar to donor communities, an effect which, for soil contact only, persisted for at least 24 h. Washing with soap and water effectively returned communities to their pre-perturbed state, although some abundant soil taxa resisted removal through washing.

**Conclusions:**

This study represents an initial characterization of bacterial relationships between humans and indoor plants, which represent a potentially valuable element of biodiversity in the built environment. Although environmental microbiota are unlikely to permanently colonize skin following a single contact event, repeated or continuous exposures to indoor biodiversity may be increasingly relevant for the functioning and diversity of the human microbiome as urbanization continues.

**Supplementary Information:**

The online version contains supplementary material available at 10.1186/s40793-022-00457-7.

## Introduction

Keeping indoor potted plants is common across the world and has recently increased in popularity [1, 2]. Indoor plants may provide various benefits, including perceived air quality improvement, interior aesthetics, and psychological and cognitive health benefits [3–7]. It has been speculated that potted houseplants may also contribute to the diversity of plant- and soil-associated microorganisms encountered by building occupants [8], which may impact the human immune system or reduce risk of chronic disorders, such as allergies and asthma [9–16]. It has been shown that plants can alter microbial community diversity and composition of indoor environments [17–19]. Yet, the effects of such environmental engineering on human-associated microbial communities remain poorly understood.

Modern lifestyles tend to decrease exposure to soil- and plant-associated microorganisms. Urbanization homogenizes the outdoor environmental microbial assemblage to which residents are exposed [20] and most people in industrialized nations spend the vast majority of their lives indoors [21], which significantly moderates their microbial exposures. Typical indoor environments include more human-associated taxa than outdoor environments [22–25] due to constant microbial shedding by building occupants [26] and filtering of outdoor microbiota by building envelopes and air filtration systems. This situation has prompted experiments with other means of increasing exposure to soil- and plant-associated microbiomes [27]. Keeping indoor plants and participating in backyard gardening and nature-based activities are hypothesized to increase interactions with soil- and plant-associated microbes [28–30], potentially providing a ’microbiome rewilding’ effect [31] or protecting against human pathogens through competitive advantage [32]. Such exposures may drive temporary or long-term changes in human skin microbial community composition, although empirical data remain scarce.

Despite their potential to affect human-associated microbiomes and hence human health, little is known about the variation of indoor plant microbiomes by plant type, source nursery, or substrate (henceforth, we use the term ’substrate’ to encompass both outdoor soil and commercial potting mix; not to be confused with ’growth media,’ which refers to substrates used in microbial cultures). Abundant research has investigated microbiome assembly and dynamics of outdoor plants, including agricultural crops, wild and urban trees and shrubs, and ornamental flowers, but only a few studies (for example, [33] and [18]) have focused on indoor plant microbiomes or whether and how potting mix microbial communities differ from those found in natural soils. Furthermore, the transmissibility of microbiota from these environmental sources and their ability to remain on human skin is poorly understood. Here we characterize the diversity of bacterial communities inhabiting the substrates and leaves of five indoor plant species, and quantify microbial transfer dynamics and residence time on human skin following simulated touch contact events with outdoor soil and leaf top surfaces. We hypothesized that (1) both soil and leaf donor microbial communities would transfer new bacterial taxa to the recipient skin community, and that these initial transfer effects would likely persist on the order of hours; (2) the effects would largely vanish within 24 h; and (3) washing with soap and water would remove the signature of the experimental transfer. With the high prevalence of indoor plants and the likelihood that people physically interact with their leaves and substrates, a basic understanding of the indoor plant microbiome may help guide interior design decisions, including the number, type, and placement of plants, that could be beneficial for building occupants.

## Methods

### Overview

In this study, we collected microbial samples from substrates and leaves of five different indoor plant types (Additional file 1: Fig. S1), and forearm skin of human subjects before and after transferring microbial propagules from outdoor soil and plant leaves. The study was performed on June 20–21 and 27–28 and July 13–14, 2016, at the Energy Studies in Buildings Laboratory (ESBL) facility in Portland, Oregon, from approximately 9:00 am–7:00 pm on the study days listed. Outdoor soil was sourced from a farm in Mohawk, Oregon, USA, while potting mix samples were taken directly from pots containing the plants used in the study; the potting mix (Gray’s Organic Potting Soil) was originally sourced from Gray’s Garden, Eugene, Oregon, USA. Indoor plant types studied were *Spathiphyllum* (Peace Lily), *Dieffenbachia* (Dumb Cane), *Dracaena* (Dragon Tree), *Sanseveria* (Snake Plant), and *Calathea* (Prayer Plant). Plants were purchased on April 10 and May 15, 2016, and were repotted into the same potting mix (Gray’s Organic Potting Soil) after purchase. Plants were kept in a climate-controlled chamber at a constant temperature of 24.4 $^{\circ }{\hbox {C}}$ and relative humidity of 42.6% until the day of sample collection. Sixteen adult subjects between the ages of 18–35 were recruited to participate in the transfer experiments. Eligibility requirements for participation in the study included (1) that the individual was in generally good health, (2) free of skin conditions or infections, and (3) had not taken antibiotics within the prior 6 months. Subjects were asked to refrain from bathing or applying topical items to the skin for a 12-h period preceding the experiment. The subjects were informed as to the full nature and design of the study and gave written consent to be participants. This study and its associated research protocols were approved by the IRB at the University of Oregon on December 23, 2013 (Reference #: 03112016.016). All researchers assigned to this protocol were CITI certified to work with human subjects.

### Baseline microbial sample collection

We used sterile nylon-flocked swabs (Copan Diagnostics; Murrieta, CA, USA) to collect microbial communities from substrates (outdoor soil, potting mix), plant leaves (top and bottom surfaces), and forearm skin. Prior to use, swabs were moistened with a sterile saline solution (0.15 M NaCl; 0.1% Tween20) and then excess moisture was removed by flicking the swab carefully. Outdoor soil was 2-mm sieved and passively air-dried prior to aliquoting subsamples; each subsample was assigned as a donor for one recipient subject for the transfer experiment. A baseline sample was collected from each outdoor soil subsample prior to performing the transfer. Potting mix samples were collected from pots containing the plants used in the transfer experiment, as well as one sample from an unopened potting mix bag. Due to qualitative differences in texture and moisture at the time of sampling, in comparison to outdoor soil, potting mix samples were not subjected to sieving or air-drying. To collect outdoor soil and potting mix samples, pre-moistened swabs were briefly dipped into a 8 mL aliquot of substrate, resulting in approximately 0.0075 g soil/mix per swab. For leaf samples, a 10 $\times$ 10 cm area of the top surface of a single healthy plant leaf from each type of indoor plant was swabbed for approximately 15 s while rotating the swab; this procedure was repeated for the bottom surface of each leaf with a different swab. Similarly, skin surface samples were collected by swabbing from standardized sampling grids measuring 1 $\times$ 3 cm drawn on each subject using ethanol-disinfected custom plastic vinyl stencils and thin-tipped permanent markers (Additional file 1: Fig. S2). Five equal and distinct grid cells were designated for sampling the skin at five time points: before transfer (0); 2-, 4-, and 8-h post-transfer; and a spot for either a 24-h post-transfer or post-wash sampling time point for each donor type; a larger area served as a donor for the skin-to-skin control (Additional file 1: Fig. S2). For the baseline skin samples, one cell was swabbed in each row of the grid, corresponding to Soil Recipient Areas, Leaf Recipient Areas, and Skin Recipient Areas at time point 0 (T00).

### Microbial propagule transfer experiment

Following collection of baseline soil, leaf, and skin samples, microbial propagules were immediately transferred from a subsample of outdoor soil, a donor plant leaf, and an adjacent area of skin to the dry inner forearm of an individual human recipient subject. Propagule transfer was accomplished by collecting donor soil/leaf/skin microbiota using the same swabbing techniques described above, then immediately applying the swab to the recipient area of skin (the entire row of the sampling grid corresponding to each donor type; Additional file 1: Fig. S2) and again rotated while swabbing firmly for approximately 10–15 s. Swabs were used in lieu of direct contact to standardize the microbial propagule size transferred to each subject from soil and leaf sources.

Only outdoor soil and top leaf surfaces of three plant types (*Spathiphyllum,* *Dieffenbachia*, and *Calathea*) were used in the transfer experiment in an effort to minimize potential sources of variability. Outdoor soil was used in the transfer experiment because (1) contact with outdoor soil is more common than contact with potting soil; (2) microbial biomass in outdoor soil is known to be high, whereas commercial potting mix is typically sterilized during manufacture; and (3) outdoor soil is frequently used to pot indoor plants. Top surfaces of leaves were used in an effort to eliminate surface location as a potential source of variation and because they are more likely to be contacted by people than bottom surfaces. A unique donor leaf was selected and swabbed (both for the baseline sample collection and for the transfer experiment) for each human subject volunteer and, similarly, a unique soil subsample was used for each subject; donor leaf/soil samples were linked with recipient skin samples for downstream analyses. Subjects S01–S06 received a leaf donor community from *Spathiphyllum*, Subjects S07–S12 received a leaf donor community from *Dieffenbachia*, and Subjects S13–S16 received a leaf donor community from *Calathea*. Skin-to-skin control transfers were performed using a designated area of dry skin on the arm to inoculate an adjacent area of dry skin. After the transfer procedure was complete, a sterile gauze dressing was lightly taped over the area with minimal occlusion and replaced between sampling periods. Subjects remained sedentary in the climate-controlled chamber for 8 h.

Swabs were also used for subsequent time-series sampling, as they have been demonstrated to be an effective method for sampling microbial diversity of the skin [34]. At each post-transfer time point, one grid cell for each donor type was swabbed. Grid columns for each time point were randomized between subjects in an effort to reduce spatial autocorrelation. To achieve both a post-wash (TW, time after wash) sampling time point and a 24-h time point (T24), we asked half of the subjects to wash the recipient skin area with Castile soap and gently pat dry with sterile paper towels just after the 8-h sampling time point for immediate re-sampling. The remaining eight subjects did not wash the recipient skin area but were sampled the following day for a 24-h post-transfer sampling time point. Between the time that the subjects left the research facility and returned the following day for the 24-h sampling time point, they were asked to refrain from thoroughly wetting the now-exposed inner forearm area, but otherwise were allowed to resume normal daily activities. For each of the three transfer types on each subject at the requisite time point 2, 4, 8, and 24-h post-transfer and post-wash, a swab sample was collected using a sterile swab (dampened with sterile saline solution: 0.15 M NaCl; 0.1% Tween20) applied to the skin and rotated while swabbing firmly for approximately 10–15 s. All baseline transfer swabs (T00) and following time-series swab samples (T02, T04, T08, T24, TW) were collected and frozen at − 20 $^{\circ }{\hbox {C}}$ for subsequent DNA extraction.

### 16S rRNA gene sequencing

A total of 331 swab samples were processed and submitted for sequencing. Negative swab, extraction kit, and polymerase chain reaction (PCR) controls were included to identify possible sources of contamination during library preparation. DNA from all samples was manually extracted using the MoBio PowerLyzer PowerSoil DNA Isolation Kit according to manufacturer’s instructions. Amplicons of the V3–V4 region (319f–806r) of the 16S rRNA gene were prepared in 50 $\upmu$L PCR reactions with one PCR step using dual-barcoded primers (see description in Supplementary Information), cleaned with Ampure beads, quantified using Quant-iT dsDNA assay kit, and pooled with equal concentrations of amplicons using an Eppendorf epMotion 5075 robot. Libraries were sequenced across two runs on an Illumina MiSeq generating 250 bp paired end reads.

Illumina sequence data were filtered, trimmed, and denoised using the DADA2 v1.5.2 statistical inference algorithm [35, 36], which identifies amplicon sequence variants (ASVs). Due to poor quality of the reverse reads, only forward reads were used. Reads were trimmed and truncated at 10 nt and 240 nt, and each read was required to have fewer than three expected errors based on quality scores. Taxonomy was assigned to ASVs using the RDP classifier implemented in DADA2 and the Silva version 132 reference database [37], with an 80% bootstrapped threshold for retaining classifications. We omitted sequence variants classified as chloroplasts or mitochondria, and those that were unclassified beyond the kingdom level. Putative contaminants were identified with decontam [38] and removed prior to downstream analyses, resulting in elimination of 71,467 reads. We also removed samples that failed to meet our minimum size threshold of 1000 reads. No significant batch effect of sequencing run was observed.

### Absolute abundance estimation with qPCR

We estimated absolute abundance as total counts of 16S rRNA gene copies per swab for donor skin, outdoor soil, and leaf top surface microbiomes using real-time quantitative PCR (qPCR; Applied Biosystems StepOnePlus System). Swabs represent approximately 0.0075 g of soil, 100 cm^2^ of leaf surface, or 3 cm^2^ of skin surface. As the primary intent of this study was to quantify changes in the skin microbial community abundance, diversity, and composition after a simulated touch contact event, rather than to compare skin microbial biomass with that of soil or plant leaves, we did not attempt to normalize samples by dry weight or surface area. The reaction mixture was prepared according to guidelines provided by ABS PowerUp SYBR Green PCR Master Mix for a ${20}\,{\upmu }\hbox {L}$ reaction and run in triplicate: ABS PowerUp SYBR Green PCR Master Mix (${10}\,{\upmu \hbox {L}}$), ${10}\,{\upmu \hbox {M}}$ Total Bacteria F SYBR Primer $5^{\prime }$-gtgStgcaYggYtgtcgtca-$3^{\prime }$ (${0.8}\,{\upmu \hbox {L}}$), ${10}\,{\upmu \hbox {M}}$ Total Bacteria R SYBR Primer $5^{\prime }$-acgtcRtccMcaccttcctc-$3^{\prime }$ (${0.8}\,{\upmu \hbox {L}}$), PCR grade water (${6.4}\,{\upmu \hbox {L}}$) and undiluted DNA template (${2}\,{\upmu \hbox {L}}$) [39]. We also used the suggested ABS PowerUP thermocycling conditions for primers with $\hbox {Tm} < {60}\,{^\circ \hbox {C}}$: initial denaturation for 2 min at ${50}\,{^\circ \hbox {C}}$, 2 min at ${95}\,{^\circ \hbox {C}}$; 40 cycles of 15 s at ${95}\,{^\circ \hbox {C}}$, 15 s at ${60}\,{^\circ \hbox {C}}$, 60 s ${72}\,{^\circ \hbox {C}}$; followed by a melt curve in the range of ${60}\,{^\circ \hbox {C}}$ to ${95}\,{^\circ \hbox {C}}$. Standard curves were generated using 10-fold serial dilutions of synthetic 167 bp gBlocks Gene Fragments (Integrated DNA Technologies, Coralville, Iowa, USA) of the same region amplified with the above primers, with known gene sequence copy numbers. To correct for differing reaction efficiencies across the different source community sample types, particularly since soil contains PCR inhibitors that reduce amplification efficiency [40], we used LinRegPCR [41] to quantify 16S rRNA gene copies from fluorescence values.

### Statistical analysis

All analyses were conducted in the R statistical programming language [42]. Differences between qPCR-based estimates of initial taxon abundances within and across groups (outdoor soil, leaf top surfaces, baseline skin) were examined using analysis of variance (ANOVA) on log-transformed corrected counts and Tukey’s post-hoc test. Estimating alpha diversity from amplicon sequencing data is an area of active research and discussion [43–46]. The Shannon diversity index, despite its wide usage, is particularly sensitive to numbers of unseen members of the true community, which are notoriously difficult to accurately estimate in environmental microbiome studies due to inadequate sampling effort relative to the true diversity of microbial communities [47, 48]. To address these limitations, we first examined sample coverage using species accumulation curves, as implemented in iNEXT [49, 50], then compared effective numbers of species (Hill numbers) based on Shannon entropy and Simpson diversity index to robustly characterize alpha diversity of pre-transfer skin, leaf, and soil samples. The iNEXT algorithm adjusts for unseen taxa by subsampling without replacement, then extrapolating from successively smaller subsamples to construct a curve representing expected alpha diversity for a given community as a function of sampling effort. This type of asymptotic species richness estimation is preferable to interpolation or rarefaction, which downsamples to the lowest sample size and discards a potentially large amount of data [51]. To quantify effects of soil and leaf propagule transfers on skin alpha diversity, we used the paired sample t-test, with the exception of the leaf transfer comparison between T00 and T02, which failed to meet requirements of normality and sphericity and was tested with the non-parametric Friedman test.

To examine beta diversity of outdoor soil, potting mix, plant leaves, and skin, we visualized clustering in a principal coordinates analysis (PCoA) ordination and performed permutational multivariate analysis of variance (PERMANOVA) using the adonis function in vegan [52] on Morisita-Horn dissimilarities. The abundance-based Morisita-Horn dissimilarity index was selected because it is robust against differences in sampling depth and undersampling [53, 54], both of which conditions are present in these data. Due to the high level of dissimilarity observed for the two types of substrates, we further investigated taxonomic differences between outdoor soil and potting mix using a negative binomial generalized linear model, as implemented in DESeq2 [55]. Degree of dissimilarity between donor communities and recipient skin communities at different post-transfer time points was tested with ANOVA using Morisita-Horn index.

We assessed persistence of specific soil- and leaf-associated bacterial taxa on skin by first selecting ASVs present in skin experimental samples after the transfer event (T02) that were also present in soil/leaf donor samples, but not present in skin baseline samples. Each skin sample was compared only with the corresponding soil/leaf donor sample; that is, samples were not aggregated by type. From this list of transferred ASVs, we filtered out those that had less than 0.001 relative abundance, sample-wise, and then visualized occurrence and abundance as a heatmap.

## Results

### Bacterial community structure of indoor plant leaves, substrates, and baseline skin samples

#### Overview

**Fig. 1:**
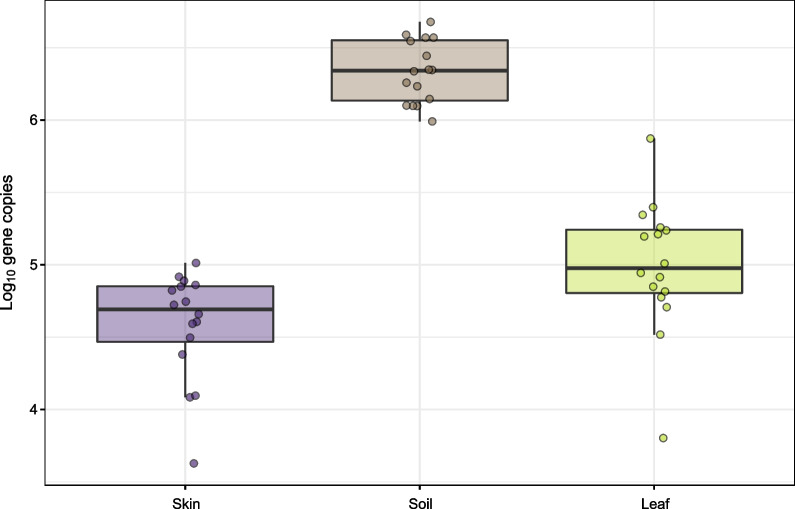
Log_10_ bacterial 16S gene copy abundances for skin, outdoor soil, and leaf samples prior to the transfer event. Note that samples were not normalized by dry weight or surface area; skin samples represent a 3 cm^2^ surface area, soil samples represent 0.0075 g of soil, and leaf samples represent a 100 cm^2^ surface area

We collected microbial samples from two types of plant substrates (outdoor soil and potting mix), leaf tops and bottoms of 14 indoor plants, representing five different genera—*Calathea*, *Dieffenbachia*, *Dracaena*, *Sanseveria*, and *Spathiphyllum*, and human skin before and after transferring soil and leaf microbiota. After quality filtering, we observed a total of 6,622,107 reads, representing 12,864 unique amplicon sequence variants (ASVs) across 22 bacterial Phyla. Outdoor soil samples had, on average, over an order of magnitude greater estimated bacterial abundance (i.e. 16S rRNA gene copy numbers) compared to leaf top surface samples (Fig. 1; Tukey’s HSD: $\beta$_soil_ = 1.3, $P < 0.005$). Prior to swab transfers, skin had lower absolute abundance of bacterial cells than either outdoor soil (Tukey’s HSD: $\beta$ = − 1.7, $P < 0.005$) or leaf top surfaces (Tukey’s HSD: $\beta$ = − 0.41, $P = 0.01$) with the caveat that samples were not normalized by dry weight or surface area and are intended to be interpreted as estimates of propagule size for the simulated touch transfer event. Potting mix and leaf bottom surfaces were not included in qPCR assays.

Substrate (outdoor soil and potting mix samples aggregated) communities were dominated by Actinobacteria (44%), Proteobacteria (25.7%), Gemmatimonadetes (19.1%), and Verrucomicrobia (3.89%). Leaf communities (top and bottom surfaces aggregated) were inhabited primarily by members of Actinobacteria (41.4%), Deinococcus-Thermus (30%), Proteobacteria (15.4%), and Bacteroidetes (8.63%). Baseline skin samples were largely dominated by Actinobacteria (65.1%), Proteobacteria (21.8%), Bacteroidetes (9.45%), and Firmicutes (2.31%).

**Fig. 2:**
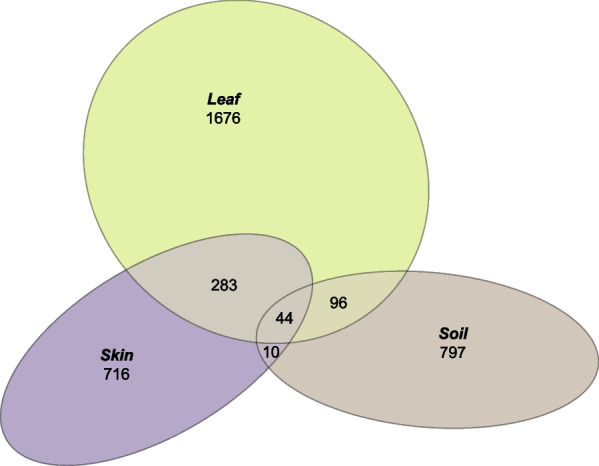
Euler diagram of unique and shared bacterial taxa for donor soil and leaf samples and pre-transfer skin samples

The most abundant ASV found in substrate samples was identified as a member of *Sphingomonas*, comprising 3.36% of all sequences; on leaves *Aeromicrobium* sp. was the most abundant ASV, comprising 4.17% of all sequences. The most abundant ASV observed in pre-transfer skin samples was *Propionibacterium* sp., comprising 44.7% of sequences. At baseline, leaf and skin samples shared 327 ASVs, while soil and skin samples shared only 54 ASVs (Fig. 2). Leaf bacterial communities had the highest number of unique ASVs, more than double the number observed in either skin or soil communities. Effectively all baseline skin samples were characterized by high relative abundances of four genera—*Streptococcus*, *Staphylococcus*, *Corynebacterium*, and *Propionibacterium*.

#### Alpha diversity

In general, sample coverage was high and species accumulation curves showed that most samples were close to the asymptotic estimates of community richness (Additional file 1: Fig. S3). Leaf samples had the highest average observed numbers of species and the widest variance, however, soil had higher effective numbers of species than leaves, based on Shannon entropy (Table 1; Tukey’s HSD: $\beta$ = 42, P = 0.01) and Simpson index (Tukey’s HSD: $\beta$ = 38, $P<0.005$). Estimates for the effective numbers of species are lower than observed richness because microbial communities are typically characterized by having a long tail of rare species, which are more difficult to detect in high-biomass communities [25]. Thus, the number of equally-abundant species needed to achieve the same Shannon entropy value as the observed community is much lower. Since leaf communities had higher numbers of observed ASVs but lower effective numbers of ASVs, we conclude that leaves tended to have greater bacterial richness but lower evenness than soil. Baseline skin samples had the lowest average observed and effective numbers of species, based on Shannon entropy (Table 1; Tukey’s HSD: $\beta$_skin-soil_ = − 87.7, $P< 0.005$, $\beta$_skin-leaf_ = − 45.7, $P< 0.005$) and Simpson index (Tukey’s HSD: $\beta$_skin-soil_ = 57.3, $P< 0.005$, $\beta$_skin-leaf_ = 19.3, $P<0.005$).

**Table 1:** Mean observed and estimated effective numbers of bacterial ASVs (based on Shannon entropy and Simpson index) of bacterial ASVs for leaf, soil, and skin samples prior to the transfer event

Type	N	Observed	Eff. Species_shan_	Eff. Species_simp_
Leaf	59	215.6 (±38.4)	63.3 (±11.7)	26.0 (±4.4)
Calathea	14	133.3 (±25.6)	37.6 (±9.3)	15.4 (±4.3)
Dieffenbachia	18	299.5 (±90.2)	94.5 (±34.5)	32.5 (±11.7)
Dracaena	7	334.3 (±212.0)	50.1 (±9.9)	26.6 (±7.8)
Sanseveria	6	206.0 (±37.3)	87.0 (±24.2)	42.1 (±13.4)
Spathiphyllum	14	134.9 (±21.9)	45.3 (±13.6)	21.0 (±7.3)
Soil	28	176.0 (±7.5)	105.3 (±5.0)	64.0 (±3.2)
Outdoor.Soil	16	179.2 (±10.9)	113.8 (±7.5)	69.0 (±4.6)
Potting.Mix	12	171.8 (±10.3)	93.9 (±4.5)	57.2 (±3.6)
Skin	47	124.8 (±7.4)	17.6 (±1.8)	6.7 (±1.1)
Female	24	134.8 (±13.1)	18.0 (±2.9)	7.6 (±1.9)
Male	23	114.5 (±6.1)	17.1 (±2.3)	5.7 (±0.9)

Standard error of the mean shown in parentheses

#### Beta diversity

**Fig. 3:**
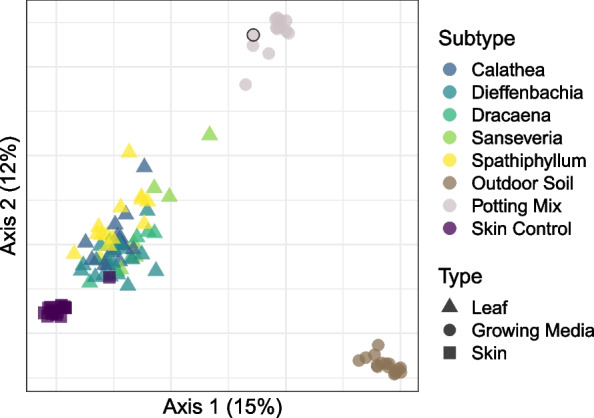
Bacterial community similarity of baseline samples collected from skin, outdoor soil, potting mix (unopened bag sample outlined in black), and leaf surfaces of five indoor plant types

Bacterial communities found in potting mix samples were compositionally dissimilar from those found in outdoor soil samples (PERMANOVA: R^2^ = 0.74, $P< 0.005$) and both were distinct from skin and plant leaf communities (Fig. 3; PERMANOVA: R^2^ = 0.21, $P < 0.005$). Despite collecting potting mix samples from pots containing plants, we did not observe an effect of plant type on community dissimilarity (PERMANOVA: R^2^ = 0.06, $P = 0.14$). Additionally, the sample from an unopened potting mix bag was indistinguishable from other potting mix samples, suggesting that the effect of watering and proximity to plant roots was minimal or overshadowed by the differences between potting mix and outdoor soil. A number of genera distinguished microbial communities in potting mix versus outdoor soil (Additional file 1: Figs. S4 and S5).

Different indoor plant genera and, to a lesser extent, individuals within the same genus tended to have different leaf surface microbiome composition (Additional file 1: Figs. S6 and S7). Even within the same individual plant (in particular, *Dieffenbachia* plant P16), samples from different leaves sometimes had wide variation in genus-level composition. In addition to the effect of plant type, leaf surface location (top vs. bottom) also had a small but significant effect on community dissimilarity, and there was a weak interaction effect with host species (Table 2). We did not detect an effect of plant nurseries on microbial community dissimilarity (PERMANOVA: R^2^ = 0.02, $P = 0.15$).

**Table 2:** Plant type and leaf surface (top vs. bottom) were significant predictors of bacterial community dissimilarity

Variable	Df	Sums of Sqs	Mean Sqs	F. Model	R2	*P* value
Plant type	4	3.73	0.93	3.10	0.17	0.0002
Leaf surface	1	1.37	1.37	4.55	0.064	0.0001
Type:Surface	4	1.50	0.38	1.25	0.07	0.024
Residuals	49	14.76	0.30	NA	0.69	NA
Total	58	21.37	NA	NA	1.00	NA

### Magnitude and persistence of microbial transfer to skin depend on source type

**Fig. 4:**
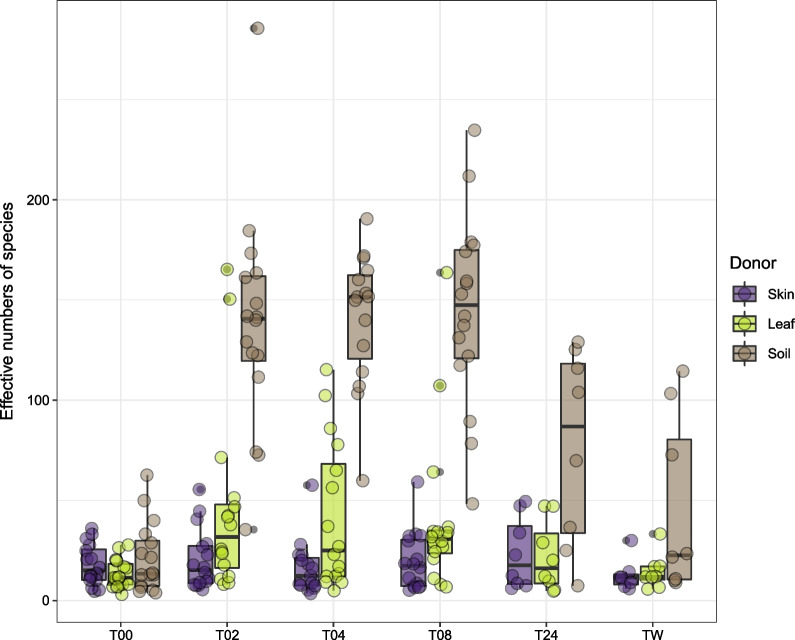
Change in alpha diversity (estimated as mean effective numbers of species) over time for skin bacterial communities after receiving transfer propagules from leaves, outdoor soil, or control skin. Effective numbers of species calculated using Shannon entropy

Alpha diversity of recipient skin receiving soil propagules increased dramatically after the transfer event (Fig. 4; paired t-test: t_15_ = − 8.1, $P<0.005$) and remained elevated for 24 h (paired t-test: t_7_ = − 3.7, $P = 0.01$). Subjects who were assigned to the hand-washing group had a substantial decrease in alpha diversity following the wash, resulting in levels that were not significantly different from baseline (paired t-test: t_7_ = − 2.0, $P = 0.08$). Recipient skin that received microbial propagules from leaves had only a small increase in alpha diversity following transfer (Friedman test: $\chi$^2^_1_ = 8.1, $P < 0.005$), which disappeared after both the wash (paired t-test: t_7_ = 0.62, $P = 0.55$) and the 24-h period (paired t-test: t_6_ = − 1.7, $P = 0.14$). Alpha diversity for skin-to-skin controls did not increase after the transfer event (paired t-test: t_15_ = − 0.94, $P = 0.36$).

**Fig. 5:**
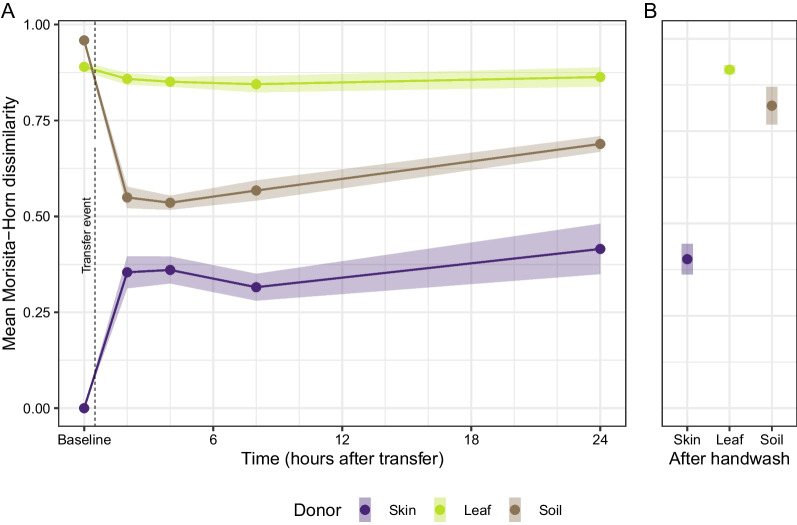
**A** Average dissimilarity (Morisita-Horn distance) of skin recipient communities at baseline (T00) and collection times following the transfer event to respective soil, leaf, and skin donor bacterial communities. Zero value indicates perfectly similar communities; value of 1 indicates perfectly dissimilar communities. **B** Subjects who participated in the soap and water wash 8 h after the transfer event had a more dramatic increase in dissimilarity than those assigned to the 24-h post-transfer collection group, suggesting that washing is more effective than time at reducing the imprint of environmental bacterial sources

Following the transfer event, community composition of skin areas that received soil microbial propagules strongly resembled donor soil compared to the pre-transfer state (Fig. 5 and Additional file 1: Fig. S8). Communities observed on the skin remained significantly more similar to soil communities, even after 24 h (ANOVA: F_1,29_ = 161.4, $P < 0.005$) and through a soap and water wash (ANOVA: F_1,29_ = 7.6, $P = 0.02$). Post-transfer samples from skin areas that received leaf microbial propagules also resembled donor communities more than baseline skin, although this change was commensurately smaller compared to the soil transfer experiments. At the 24-h census, leaf recipient skin communities were not more similar to donor communities than baseline skin (ANOVA: F_1,27_ = 3.0, $P = 0.10$). After washing, recipient skin was more dissimilar from leaf donors than baseline skin (ANOVA: F_1,27_ = 10.5, $P = 0.01$). Control skin samples, which received propagules from an adjacent area of skin, became less similar to the initial pre-transfer (T00) sample over time, possibly due to spatial variation.

In total, 291 ASVs found in soil donor samples, but not observed in baseline skin samples of the corresponding individual, were enriched on the skin 2 h after the soil transfer event (Additional file 1: Fig. S9). Many of these persisted at least 8 h post-transfer, and some were not completely removed even after washing or after a 24-h period. In contrast, few ASVs associated with leaf donor samples were substantially enriched on skin at the 2-h or later censuses (Additional file 1: Fig. S10).

## Discussion

It has been suggested that modifying the indoor microbiome through ’bioinformed design’ [56] could help promote human well-being by engineering exposures to potentially-beneficial microorganisms. In particular, built environments that are highly controlled and sanitized, such as hospitals and cleanrooms, may experience a loss of microbiome diversity and development of antibiotic resistance as a result of stringent cleaning regimes [57]. One possible avenue for re-establishing indoor microbial diversity is through the addition of houseplants [8, 17, 18]. However, characterizing the innate microbiome of houseplants is a necessary first step towards achieving such goals. This study represents an initial effort to describe the microbial communities associated with leaves and substrates of several common houseplants, as well as their ability to transfer to human skin.

### Houseplant microbiome characteristics

In this study, we found that community composition of outdoor soil was distinct from potting mix, an effect driven by the enrichment of 40 bacterial taxa, including *Arthrobacter*, *Gaiella*, *Gemmatimonas*, *Massilia*, and *Sphingomonas* in soil samples, and enrichment of 33 other taxa, including *Actinospica*, *Gordonia*, *Hyphomicrobium*, *Mucilaginibacter*, *Nocardia*, and *Rhodanobacter*, in potting mix (Additional file 1: Figs. S4 and S5). Interestingly, close relatives of many taxa enriched in potting mix are known to inhabit acidic, nutrient-deficient, and/or polluted soil environments [58–60]. *Rhodanobacter* and *Hyphomicrobium*, in particular, have been previously recovered from potting media [61, 62], sometimes associated with *Spathiphyllum* plants where they may play a protective role against root-rot pathogens [63]. Some researchers have suggested that microorganisms abundant in houseplant substrates are responsible for VOC removal in living biofilters, due to their ability to degrade a wide range of organic pollutants [62].

Indoor plant leaf microbiomes characterized in this study were dominated by members of Actinobacteria, Deinococcus-Thermus, Proteobacteria, and Bacteroidetes, which are the same highly abundant phyla (with the exception of Deinococcus-Thermus) typically present in the phyllosphere in outdoor settings [64–66]. Genera commonly found on plant leaves in outdoor studies, such as *Pseudomonas*, *Sphingomonas*, *Methylobacterium*, *Bacillus*, *Massilia*, *Arthrobacter*, and *Pantoea* [67], were also highly abundant in this study, though several genera appeared to be preferentially associated with certain types of indoor plants (Additional file 1: Fig. S7). Specifically, we noted that genus *Aurantimonas* was enriched on *Calathea* plants, *Devosia* on *Dieffenbachia*, and *Methylobacterium* on *Spathiphyllum* plants. Close relatives of these bacterial genera are known for their stress-resistance [65, 68], allowing them to inhabit harsh environmental conditions, such as those generally ascribed to leaf surfaces. A speculative explanation for the greater richness but lower evenness in leaf samples compared with substrate samples is the likelihood of more transient taxa (i.e., taxa deposited from air) present on leaf surfaces than in the substrate matrix. This is a likely possibility, since some plant leaves were visibly dusty prior to sampling. Previous surveys of indoor air have found up to 10^6^ bacterial cells per m^3^, roughly similar to bacterial abundance in outdoor air [69]. Alpha diversity of indoor air may be lower [69, 70], equal [71], or higher than outdoor air [72], depending on building type and ventilation system. Studies focusing on indoor dust have found between 10^3^–10^6^ bacterial particles (variously quantified as gene copies, genomes, or CFUs) per mg of dust [25, 73, 74] and greater alpha diversity than outdoor dust [75]. Thus, deposition from indoor air is expected to be a significant source of the diversity observed in leaf samples.

### Transfer and persistence on human skin

Alpha diversity of skin receiving soil microbial propagules increased immediately after the transfer event and remained elevated for at least 24 h, though not through washing; effects of leaf propagules were much less pronounced and did not last as long. These effects are likely a function of donor community absolute abundance, as Shmida and Wilson [76] stated that mass effects will always increase alpha diversity.

Community similarity between donor propagules and skin also increased following the transfer event, again, more so for soil transfer than for leaf. This may be partly explained by a combination of the initial dissimilarity of donor microbial communities to baseline skin communities and donor propagule absolute abundance. Soil microbial communities were highly distinct from those inhabiting baseline skin (Fig. 3) and also had well over an order of magnitude greater absolute abundance (Fig. 1), whereas leaf microbial communities were much more similar to baseline skin and had only marginally greater absolute abundance. We speculate that leaf and skin microbiomes were more similar to each other than to soil because they shared many similar characteristics, such as being relatively dry and exposed to air and UV radiation. Both the indoor plants and human subjects in this study spent much of their time inside buildings and, therefore, may have harbored many transient bacterial taxa associated with indoor air. In contrast, the soil used for simulated touch contact events was collected directly from a farm approximately 180 km away.

Of the 291 ASVs that were transferred from soil to skin, a number of genera, including *Arthrobacter*, *Bradyrhizobium*, *Gaiella*, *Massilia*, *Mycobacterium*, and *Sphingomonas*, remained detectable on the recipients’ skin for the entire 24-h study period, through resumption of daily activities and even a soap and water wash (Additional file 1: Fig. S9). On average, the relative abundance of taxa on skin post-transfer was correlated to their relative abundance in the donor community, indicating that, for many taxa, transfer occurs proportionally to relative abundance in the donor community. This finding is consistent with existing theoretical frameworks of metacommunity ecology and invasion biology; namely, the mass effects paradigm in metacommunity ecology, which describes the ability of organisms to establish in sites where they cannot self-maintain [76], and a parallel concept in invasion biology—propagule pressure [77]. Most exogenous taxa remained on human skin for only several hours, whereas a small number were detected on subjects for at least 24 h, dependent on their abundance in the donor community. Similarly, other research has indicated that the probability of successful colonization by microorganisms from environmental sources depends largely on their absolute abundances in source habitats [78, 79]. Ultimately, washing the recipient area with soap and water proved most effective at removing the imprint of donor bacterial propagules. Both of these results are consistent with prior research demonstrating that direct skin contact with soil leads to a substantial, but transient, alteration of skin microbiome diversity and composition [80]. The practical effect of this is that the propagule pressure for abundant taxa emigrating to the skin is higher; on longer time scales, this implies that the probability of colonization (as distinct from the probability of acquisition, studied here) is likely to be higher for those taxa that maintain high abundance in source communities that are often contacted [81–83].

### Relevance

Touch contact with substrates such as soil or plants, which bear their own complex assemblages of microbial residents, may lead to the wholesale transmission of microbial communities, a phenomenon known as community coalescence [84]. It is possible that regular acquisition through dispersion (e.g., contact) events and the transient dynamics of resetting the microbiome are enough to explain the increased variation observed on the skin relative to the gut and oral microbiomes [85]. That is, the skin microbiome may be always in a non-equilibrium state, ‘recovering’ from a recent coalescence event. Differential persistence through time for different microbes (potentially driven by differential abundance in the source communities) means that there will be complex behavior resulting from the interaction of different time scales: the time scales on which microbes are lost (which vary by microbe, and by other disturbance events like washing or skin sloughing), and the time scales on which new contact events happen. Thus, explaining microbial community composition of skin at a given moment must take into account, not only long-term commensal microbiota, but also the transient dynamics of multiple, interacting coalescence events—the current microbial assemblage is the result of the combination of multiple immigration events in time, with each microbe acquired having its own particular decay curve, all of which must be considered together to explain any given assemblage.

Although environmental microbiota are unlikely to permanently colonize skin [86], they may be able to indirectly influence human health by interacting with commensal skin microbiota, which can then modulate the immune system [87], or even perform the same role directly [88]. Given the observed importance of source biomass on the transfer and persistence of environmentally-acquired microbiota, these results suggest that continued study of hand hygiene [89] and substrates commonly encountered in the built environment [22, 90–95] may help promote safe and salutogenic buildings in the future.

## Conclusion

Exposure to indoor biodiversity is an increasingly relevant topic as human populations continue to urbanize and individuals spend increasingly more of their lives inside buildings. In addition to their well-researched beneficial effects on mental well-being, indoor plants may also interact with human health by contributing soil- and plant-associated microbial diversity to indoor environments. However, much remains to be understood about the microbial communities inhabiting houseplants and their substrates, as well as their potential interactions with our own commensal microbiomes. This study characterized the microbial communities of several common houseplants and is one of the first culture-independent studies comparing microbial communities of outdoor soil and manufactured potting mix. It also provides a first glimpse of the bacterial relations between humans and houseplants.

## Supplementary Information

**Additional file 1**. Supplementary information.

## Data Availability

The 16S rRNA raw sequence data have been deposited in the NCBI SRA database under accession number PRJNA767119. All other data used in this study have been deposited in the open access data repository Figshare (DOI: 10.6084/m9.figshare.16655140).
