# Supplementary material for: Temporary establishment of bacteria from indoor plant leaves and soil on human skin
Source: Environ Microbiome. 2022 Dec 26;17:61. doi: 10.1186/s40793-022-00457-7 (PMC9793532; doi:10.1186/s40793-022-00457-7)
Supplement: Supplementary file 1 — Additional file 1. Supplementary information. [file 40793_2022_457_MOESM1_ESM.pdf]

Temporary establishment of bacteria from indoor plant leaves and soil on human skin

Supplementary Information

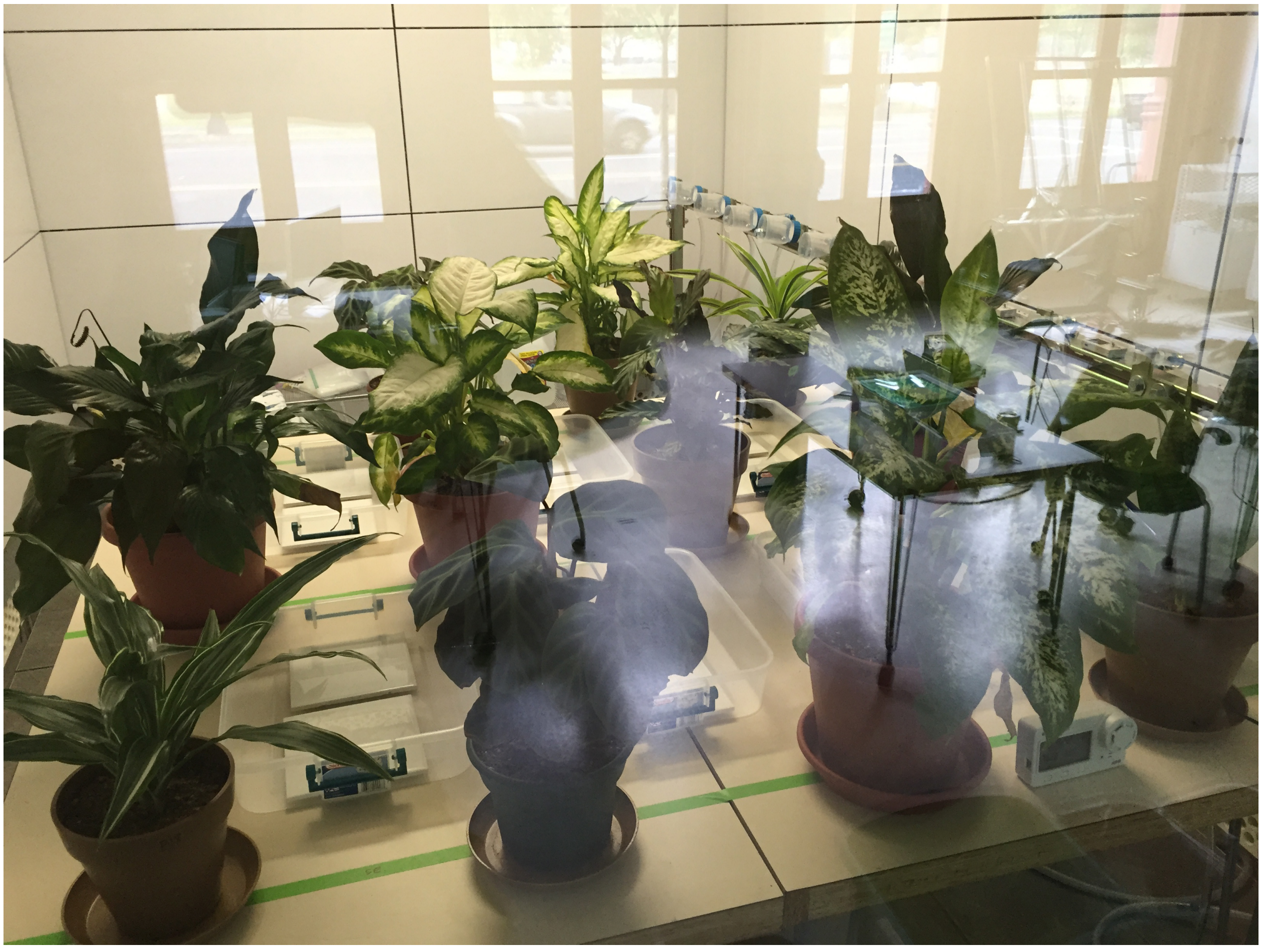

Figure S1. Indoor potted plants (*Calathea*, *Dieffenbachia*, *Dracaena*, *Sanseveria*, and *Spathiphyllum*) kept in climate-controlled chamber during the study.

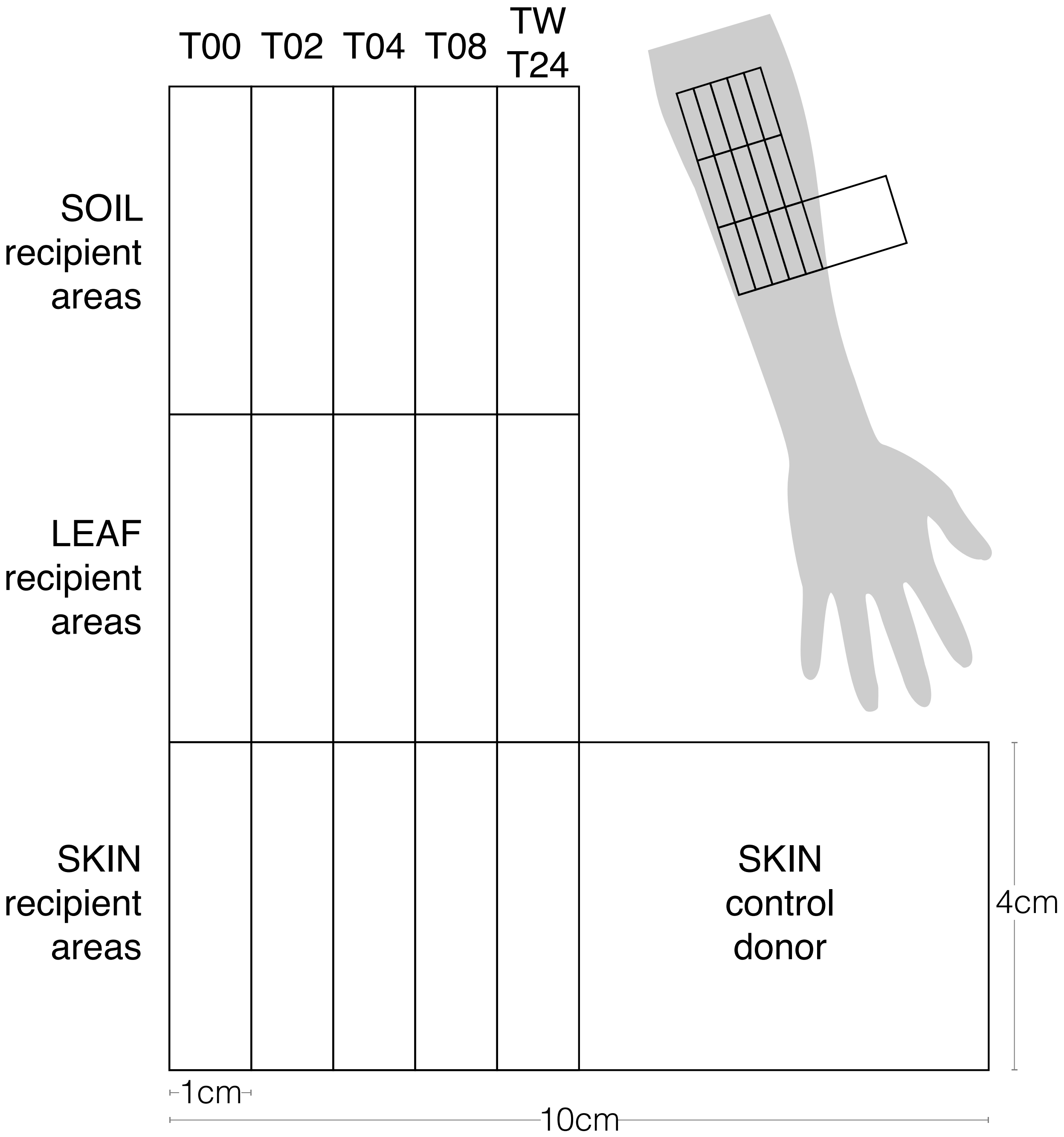

Figure S2. Schematic of sampling grid. Grid columns for each time point (0, 2, 4, and 8 hour, as well as the final 24 hour or wash sample) were randomized between subjects to avoid spatial autocorrelation.

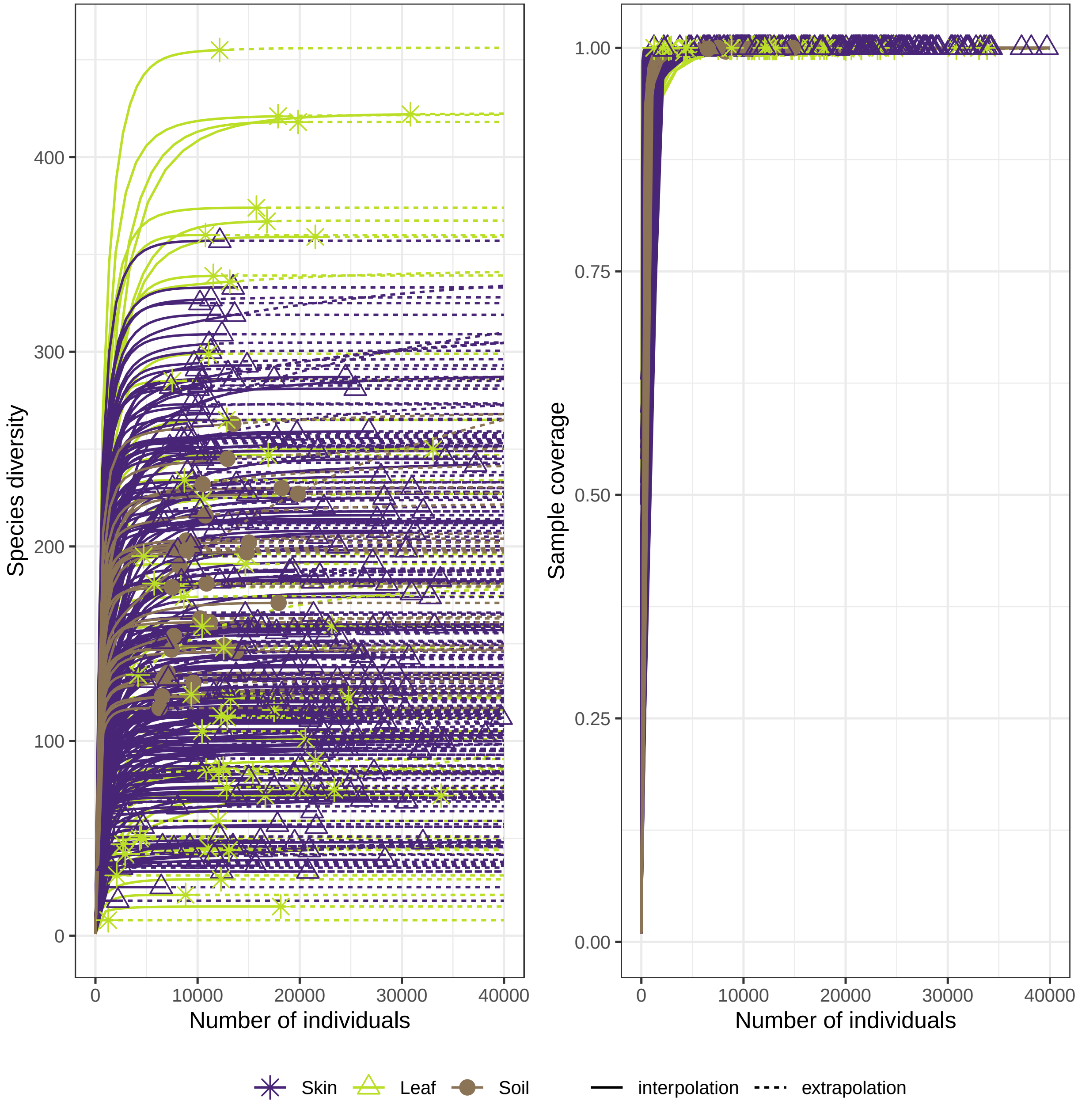

Figure S3. Rarefaction/extrapolation curve and sample completeness curve for all skin, leaf, and growing media samples in this study.

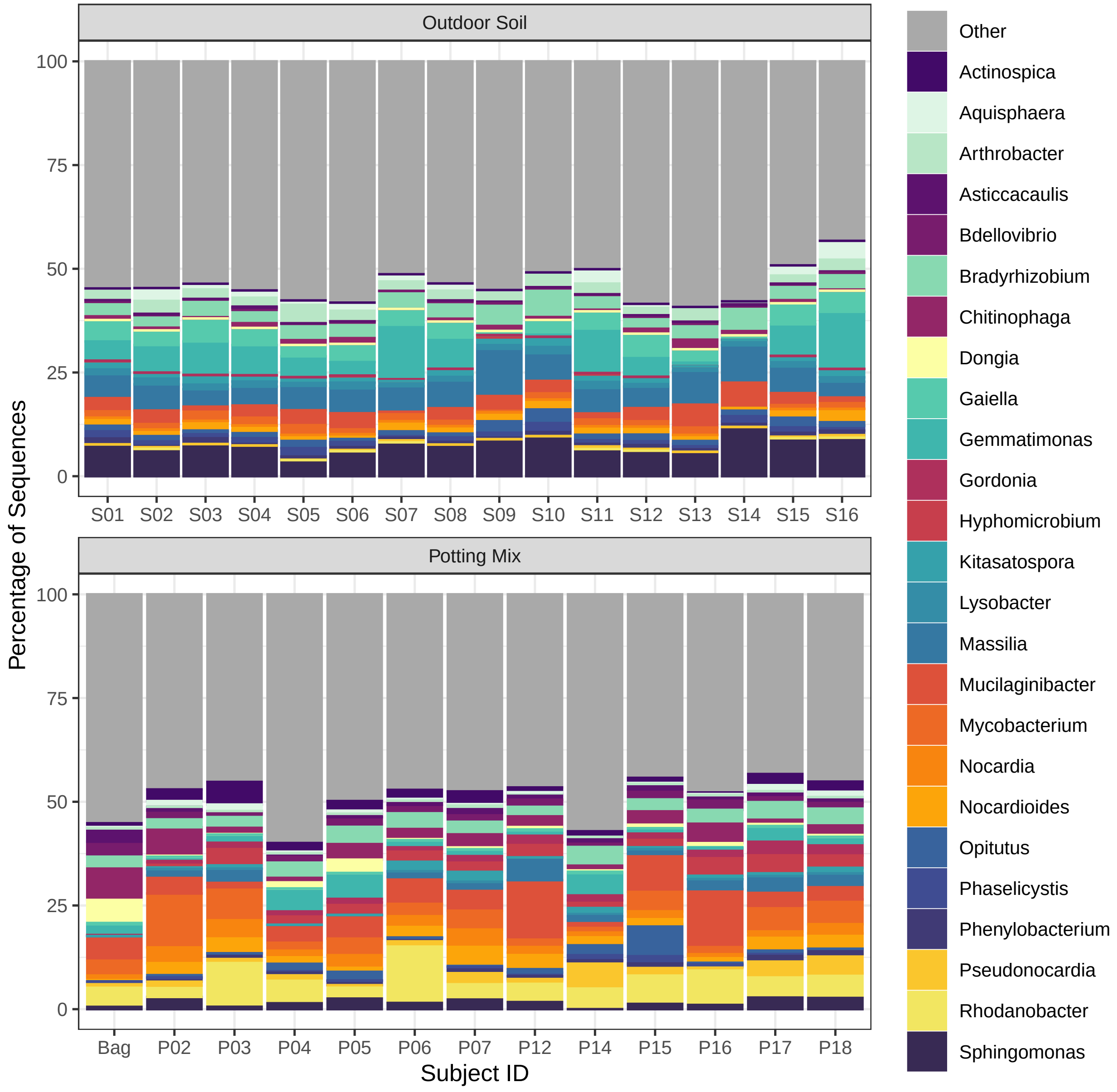

Figure S4. Genus-level composition of outdoor soil and potting mix bacterial communities. Only the 25 most abundant taxa are colored; all others are combined into the category "Other."

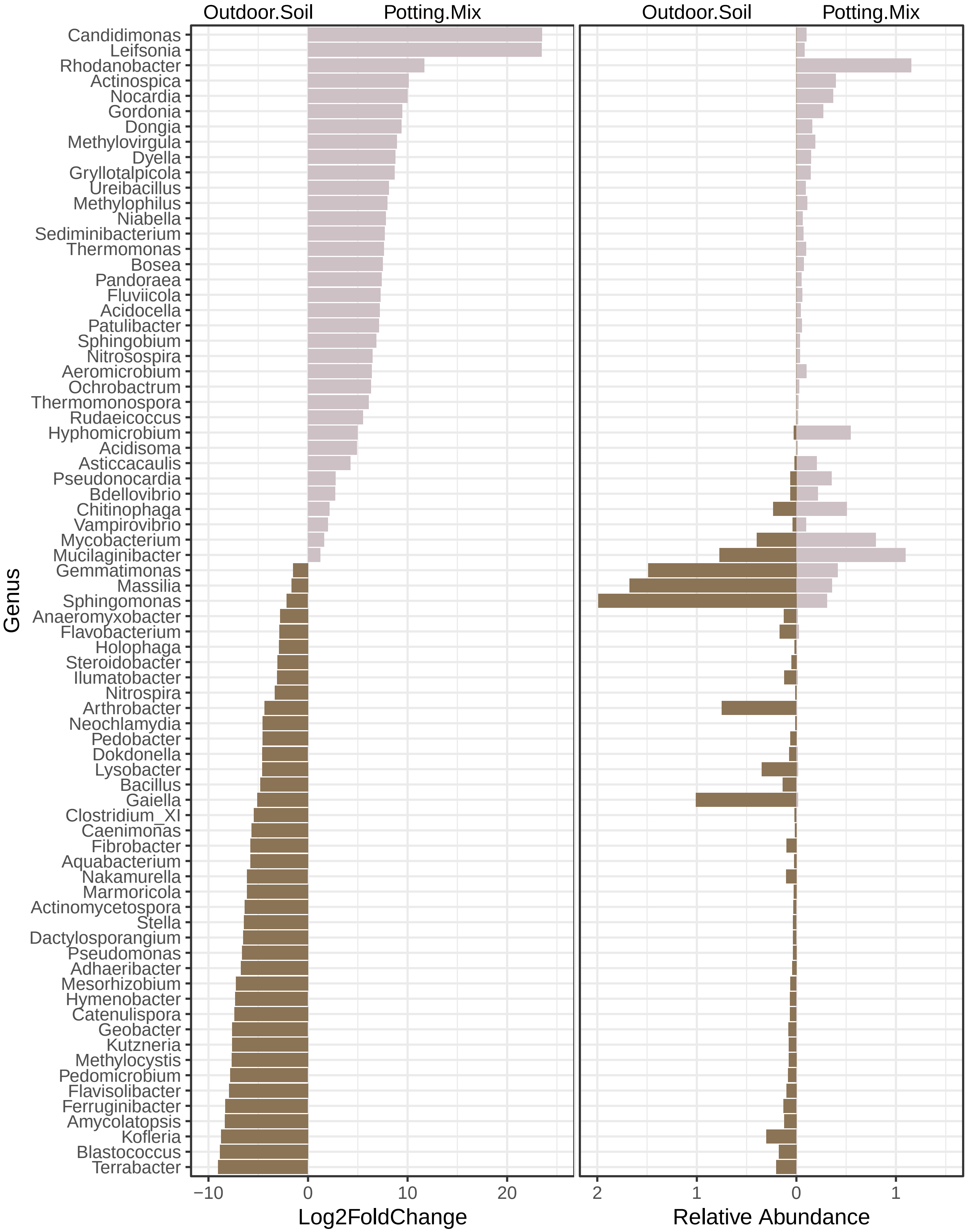

Figure S5. Bacterial genera enriched in outdoor soil versus potting mix.

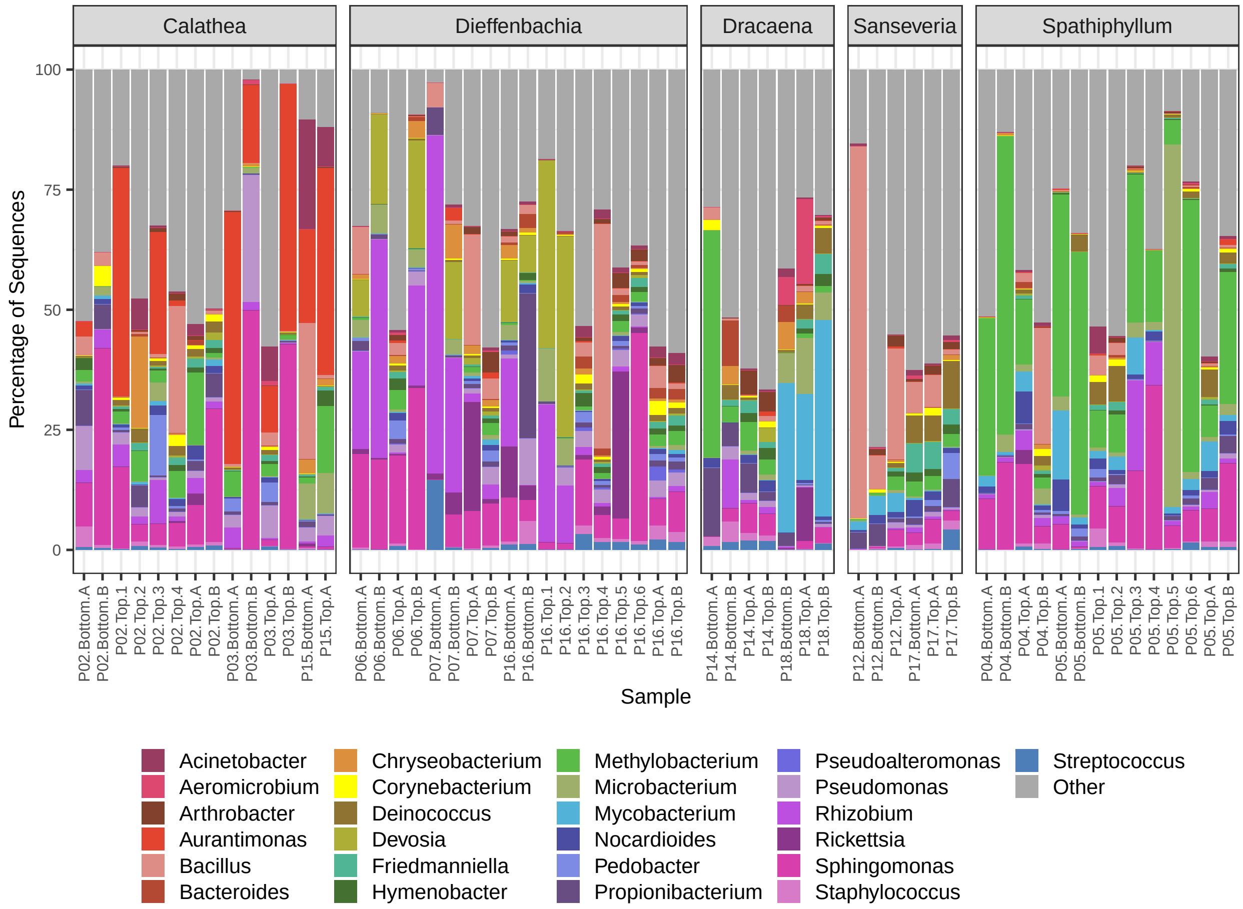

Figure S6. Genus level composition of leaf bacterial communities for the five indoor plant types included in this study. Only the 25 most abundant taxa are colored; all others are combined into the category "Other."

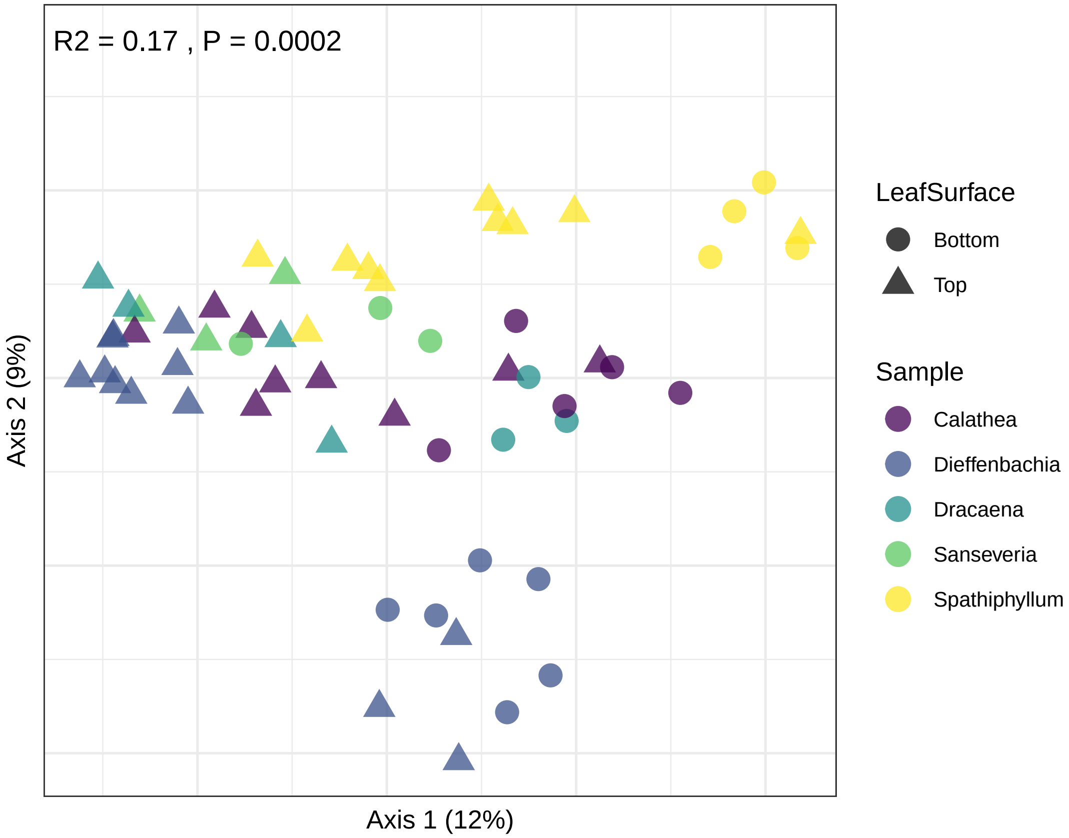

Figure S7. Dissimilarity in indoor plant leaf bacterial communities.

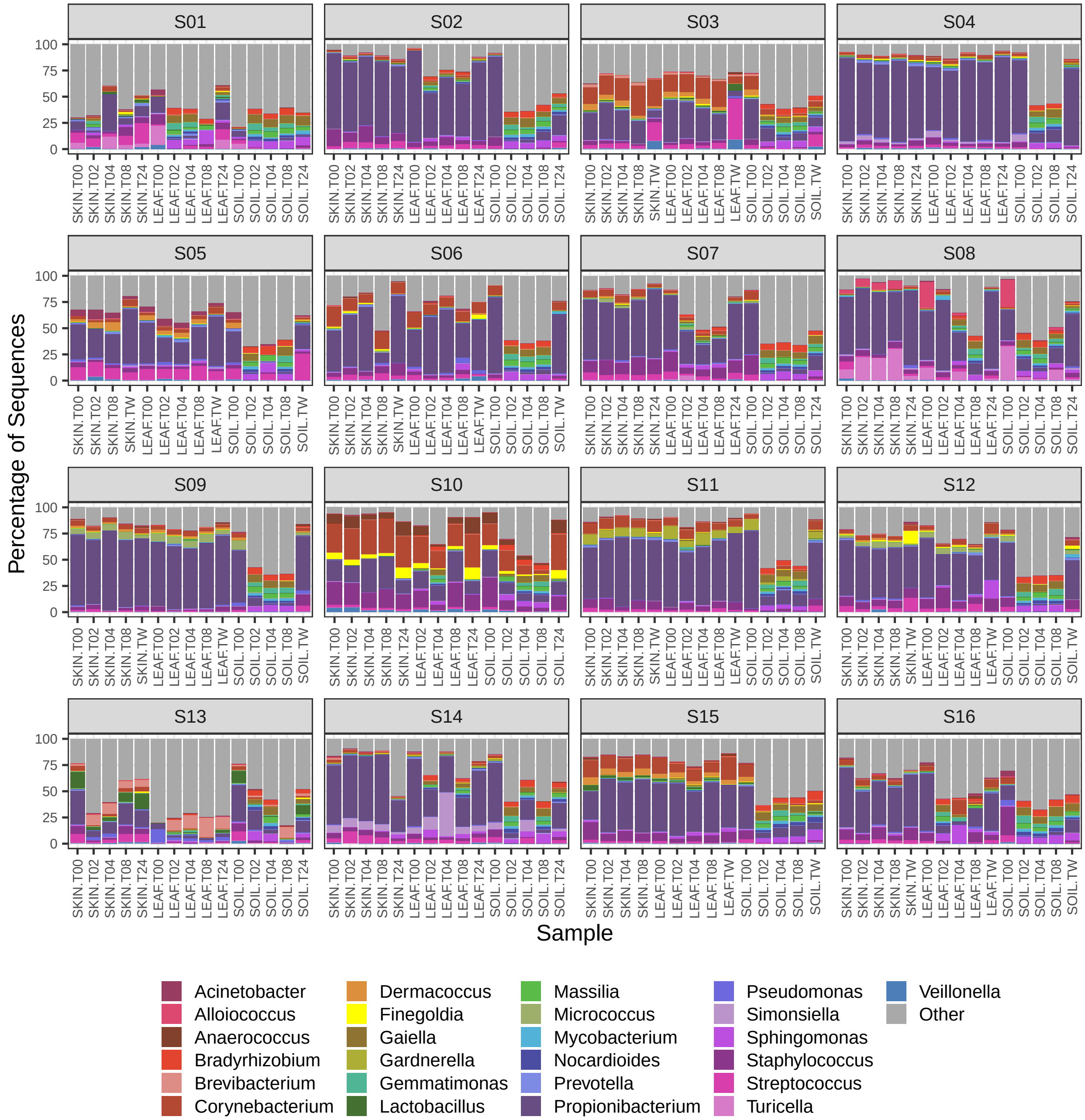

Figure S8. Skin bacterial community composition for each of the 16 human subjects in the study. The 25 most abundant bacterial genera are colored and less abundant taxa are combined into category "Other." Individual samples for each subject are organized by propagule donor (Control Skin, Leaf, and Soil) and within each donor category samples are further organized by collection timepoint (T00, T02, T04, T08, TW/T24).

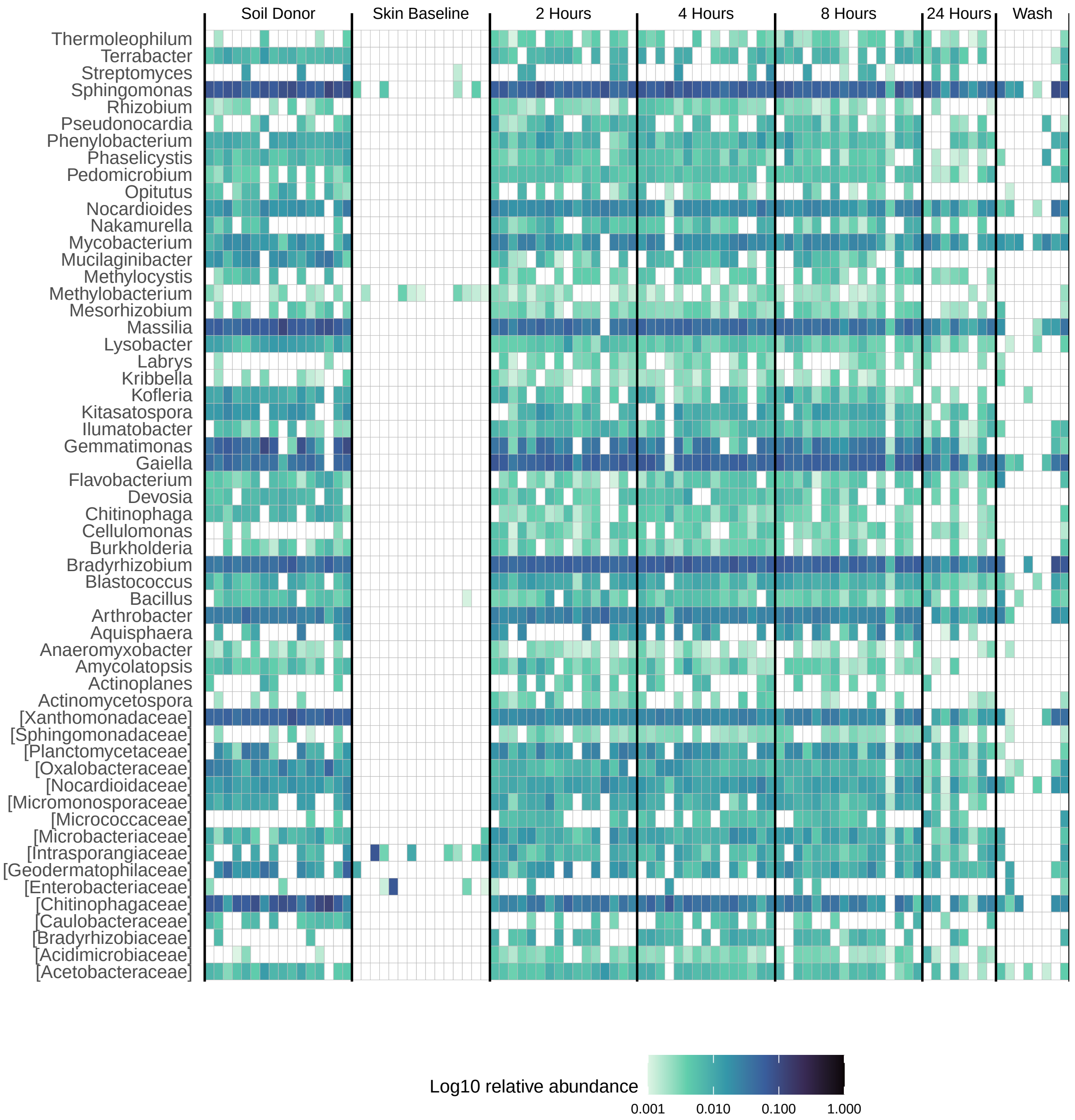

Figure S9. Relative abundance of taxa found in soil donor samples, but not on skin at baseline, that were enriched on skin after the transfer event.

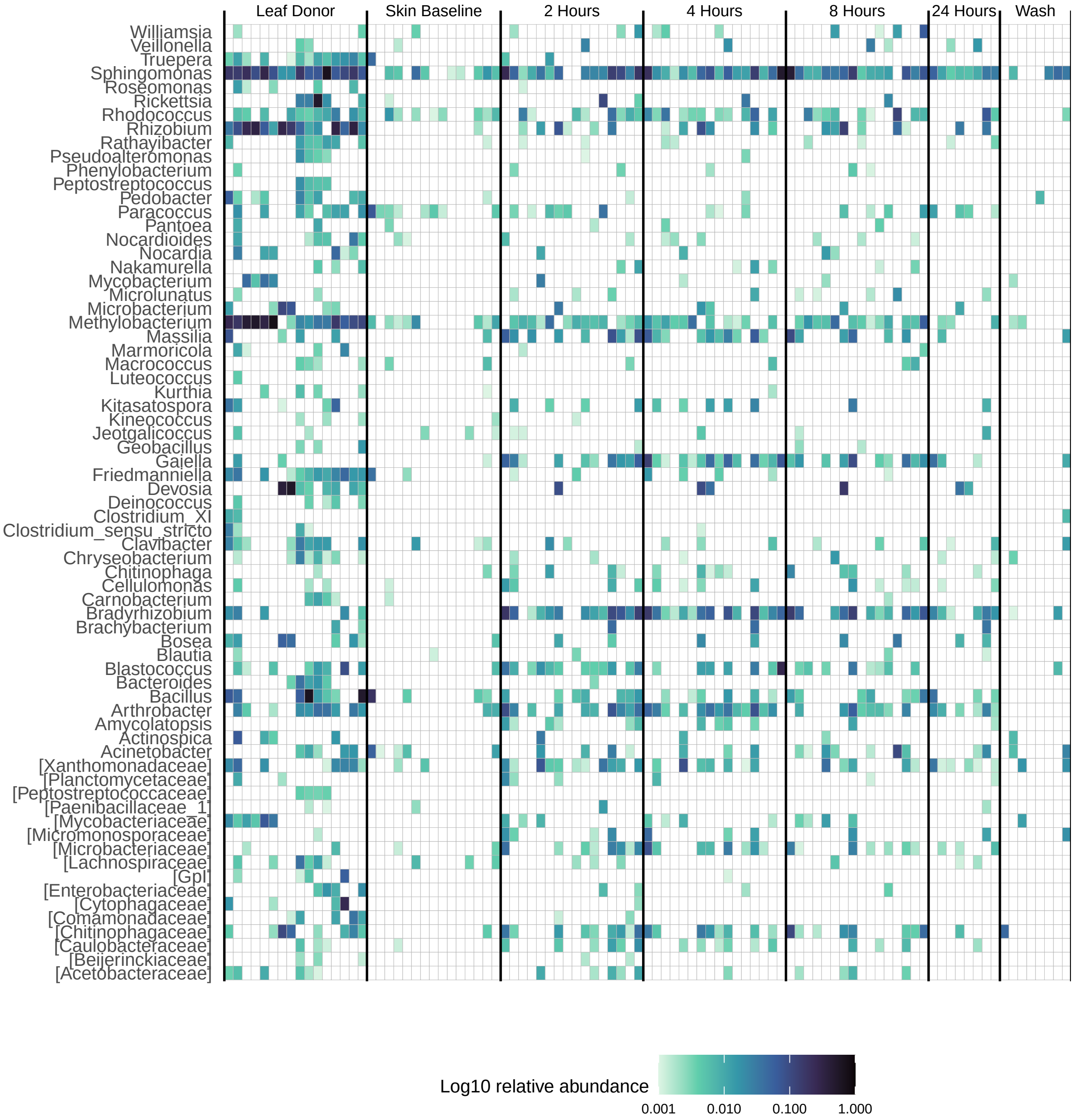

Figure S10. Relative abundance of taxa found in leaf donor samples, but not on skin at baseline, that were enriched on skin after the transfer event.
